# A fast algorithm for genome-wide haplotype pattern mining

**DOI:** 10.1186/1471-2105-10-S1-S74

**Published:** 2009-01-30

**Authors:** Søren Besenbacher, Christian NS Pedersen, Thomas Mailund

**Affiliations:** 1 Bioinformatics Research Center, University of Aarhus, Denmark; 2 Department of Computer Science, University of Aarhus, Denmark

## Abstract

**Background:**

Identifying the genetic components of common diseases has long been an important area of research. Recently, genotyping technology has reached the level where it is cost effective to genotype single nucleotide polymorphism (SNP) markers covering the entire genome, in thousands of individuals, and analyse such data for markers associated with a diseases. The statistical power to detect association, however, is limited when markers are analysed one at a time. This can be alleviated by considering multiple markers simultaneously. The *Haplotype Pattern Mining *(HPM) method is a machine learning approach to do exactly this.

**Results:**

We present a new, faster algorithm for the HPM method. The new approach use patterns of haplotype diversity in the genome: locally in the genome, the number of observed haplotypes is much smaller than the total number of possible haplotypes. We show that the new approach speeds up the HPM method with a factor of 2 on a genome-wide dataset with 5009 individuals typed in 491208 markers using default parameters and more if the pattern length is increased.

**Conclusion:**

The new algorithm speeds up the HPM method and we show that it is feasible to apply HPM to whole genome association mapping with thousands of individuals and hundreds of thousands of markers.

## Background

Identifying the genetic causes of common diseases has long been an important research area in genetics. Where early studies were limited to studying few genes at a time, due to economical and technological constraints, development in genotyping technology has revolutionised the field. It is now cost effective to obtain hundreds of thousands of genotype markers in thousands of individuals for a single study. This makes it possible to scan the entire genome for disease associated markers in a single analysis and such genome-wide association studies have recently lead to a virtual flood of newly discovered disease genes [1-7].

Most studies search for disease association through a marker-by-marker approach where each marker in turn is tested for association to the disease phenotype, e.g. using a simple Fisher's exact test or a *χ*^2^-test. However, a marker by marker approach is limited in statistical power due to the indirect testing for association, where so called "tag SNPs" are used as proxies for unobserved markers, but by using multiple markers, this problem can be alleviated [8,9]. A tradeoff must be made between method sophistication and computation efficiency when developing multi-marker approaches, however.

The Haplotype Pattern Mining method is a multi-marker approach introduced in 2000 by Toivonen *et al. *[10,11]. It is based on the idea of extracting local haplotype similarities and locating areas where haplotypes are correlated with the disease phenotype. Compared to methods based on statistical sampling [12-17] HPM is computationally much more efficient, similar to other heuristic approaches [18-20] capable of analysing genome-wide datasets. In this paper, we develop a faster version of HPM and show that it scales to genome-wide association studies.

## Methods

The goal of association mapping is to find disease-predisposing regions of the genome. This can be done by looking for differences in the frequency of genetic variants between cases and controls. Since genome sequencing is expensive the whole genomes of the case and control individuals in a case-control study are usually not sequenced. Instead only single base pairs that are known to frequently differ between humans, called SNP markers, are sequenced.

### The association mapping problem

If *k *SNP markers are typed then we can represent a chromosome by a haplotype vector H of length *k*, where *H *= (*h*_1_,..., *h*_*k*_) and *h*_*i *_∈ *alleles*(*i*) for all *i*, 1 ≤ *i *≤ *k*; *alleles*(*i*) is the domain of the *i*th marker. The input to an association mapping method then consists of a set *A *= {*A*_1_,..., *A*_*p*_} of disease-associated haplotypes and a set *C *= {*C*_1 _... *C*_*q*_} of control haplotypes.

### Haplotype pattern

A haplotype pattern P over *k *markers is a vector (*p*_1 _... *p*_*k*_), where *p*_*i *_∈ *alleles*(*i*) ∪ {*} for all *i*, 1 ≤ *i *≤ *k*, where * is the "dont't care" symbol. The haplotype pattern occurs in a given haplotype vector (chromosome) *H *= (*h*_1_,... *h*_*k*_) if either *p*_*i *_= *h*_*i *_or *p*_*i *_= * for all *i*, 1 ≤ *i *≤ *k*. The length of a pattern is defined as the maximum distance between two non-"*" characters in the pattern. Gaps are subsequences of "don't care" symbols in a pattern that are surrounded by non-"*" characters on both sides. Since long patterns are not likely to exist we only want look at a subset of the possible patterns. We call the patterns that we want to consider for legal patterns. A pattern is legal if the pattern length is less than the parameter *l*, it contains fewer than *g *gaps, and no gaps are longer than *s*.

### Strongly associated pattern

The signed *χ*^2 ^measure ± *χ*^2^(*P*) of a haplotype pattern P is the standard *χ*^2 ^measure where the sign is positive if the relative frequency of *P *is higher in cases than in controls, and negative otherwise. Given a positive association threshold *x*, we say that *P *is strongly associated with the disease if ± *χ*^2^(*P*) ≥ *x*.

### The HPM problem

Given a set of case haplotypes *A *= {*A*_1_,..., *A*_*p*_} and control haplotypes *C *= {*C*_1 _... *C*_*q*_} the goal of the HPM algorithm is to find all strongly associated patterns that are legal.

### Localizing disease genes using HPM

**Figure 1:**
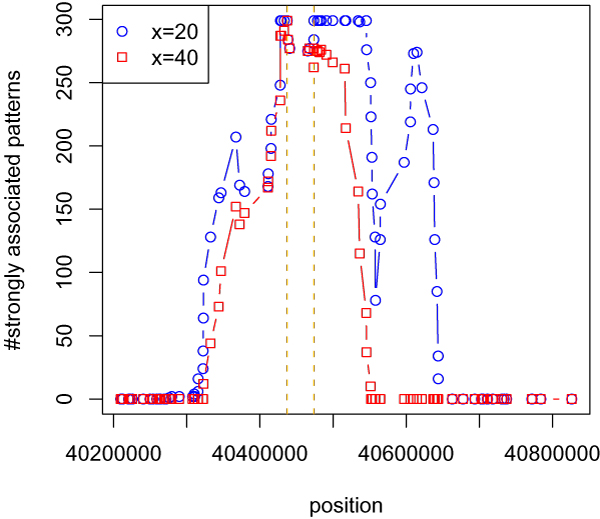
**Example of localization**. Example of localization of susceptibility alleles using HPM. The plots show the number of strongly associated patterns each marker was included in for two different values of *x*. The rest of the parameters were fixed at their default values (*l *= 7, *g *= 2, *s *= 2). The two vertical lines show the location of the two SNPs in the region that has been validated through replication.

Haplotype patterns close to a susceptibility locus are likely to be more associated with the disease than patterns further away. If we have found all strongly associated patterns we can score each marker by counting the number of times that it is contained in a strongly associated pattern. The HPM point prediction is then the marker that is most frequently contained in the strongly associated patterns. Fig. 1 shows an example of the localization of a validated susceptibility allele in the Crohn's disease data set from the Wellcome Trust Case-Control Consortium (WTCCC) [4].

#### Old algorithm

The algorithm presented in [11] recursively generates haplotype patterns using a depth-first-search strategy. To avoid looking at all possible patterns the algorithm prunes away parts of the search tree based on a lower bound on the number of disease-associated chromosomes that match a pattern.

**Figure 2:**
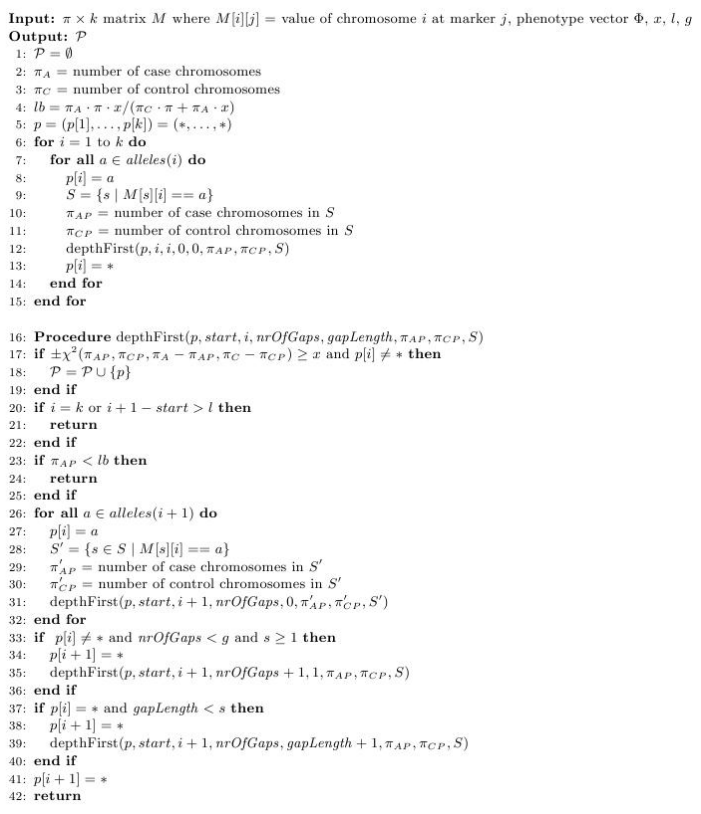
**Original HPM algorithm**. Pseudo code for the original HPM algorithm with some improvements.

Some simple improvements can be made to this algorithm. As presented in the paper counting the number of affected and unaffected individuals that match the pattern in each call to *depthFirst *will take time *O*(*n*·*l*) where *n *is the number of individuals and *l *is the length of the pattern. If we remember which individuals match the pattern at a given time then we only need to look through these when a new non-"*" symbol is inserted in the pattern. Pseudo code for the algorithm with this improvement is shown in fig. 2. The improvement greatly speeds up the algorithm.

#### New algorithm

The idea of the new algorithm is to exploit that LD structure means that you usually only see a handful of the 2^*n *^possible haplotypes if you look at *n *neighboring SNPs. Instead of looking at all different haplotype patterns spanning a region we look at all combinations of haplotypes over the region. We search these haplotype sets in a depth-first-search but stop examining a branch if there is no legal haplotype pattern that could occur in all of the haplotypes in the current set.

### Induced pattern

Given a set of haplotypes *h*_1 _... *h*_*k *_the induced pattern of the set is the haplotype pattern that occurs in all of the haplotypes and contains fewest possible "*"("don't care") symbols.

An induced pattern over a set of haplotypes that is not legal can sometimes be made legal by inserting extra "*" symbols if *s *> 2. This happens if a pattern is illegal because it contains too many gaps but would become legal if two gaps were joined into one. If for example *l *= 5, *g *= 1 and *s *= 3 then "0 * 1 * 0" is an illegal pattern because it contains more than *g *gaps. The pattern can however be made legal by substituting the "1" for a "*" yielding the pattern "0 * * * 0".

### Valid pattern

An induced pattern over a set *S *of haplotypes is said to be valid with regard to *S*, if the pattern occurs in all of the haplotypes in *S *but not in any of the other haplotypes found in the input data.

### Equivalent pattern

A haplotype pattern will split the set of individuals into those that match the patterns and those that do not. We say that two patterns are equivalent if they result in the same bipartitions of the set of individuals.

### The algorithm

**Figure 3:**
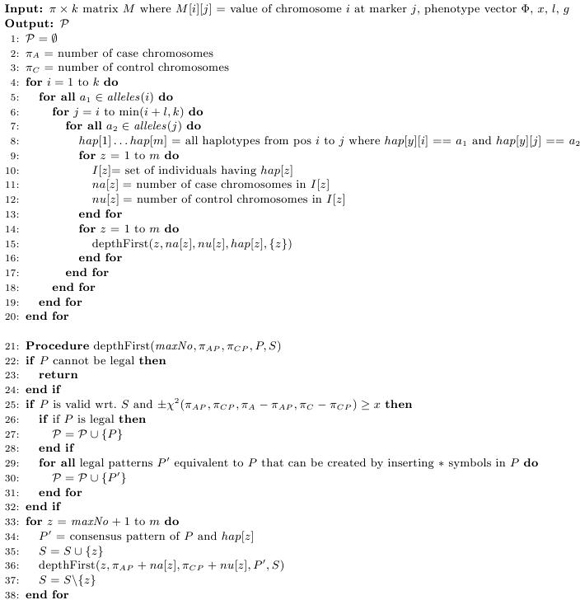
N**ew HPM algorithm**. Pseudocode for the new HPM algorithm.

The new algorithm (Fig. 3) looks at sets of haplotypes. It traverses all possible combinations of haplotypes by gradually expanding a set one haplotype at the time. If at any point the induced pattern of the current set of haplotypes cannot be made into a legal pattern by adding extra "*"-characters the current set is not expanded further. If a pattern is valid and strongly associated it is added to the output set along with all its equivalent patterns.

## Results and discussion

We have implemented both the old and the new algorithm in Python using the *SNPfile *library [21] to read and store the input data. To evaluate the algorithms, we have used the Crohn's disease data set from the Wellcome Trust Case-Control Consortium (WTCCC) [4]. This data set contains 491208 markers in 2005 disease affected individuals and 3004 unaffected control individuals. We used the *Beagle *[22] program to phase the haplotypes and infer missing genotypes.

### Time vs. number of individuals

**Figure 4:**
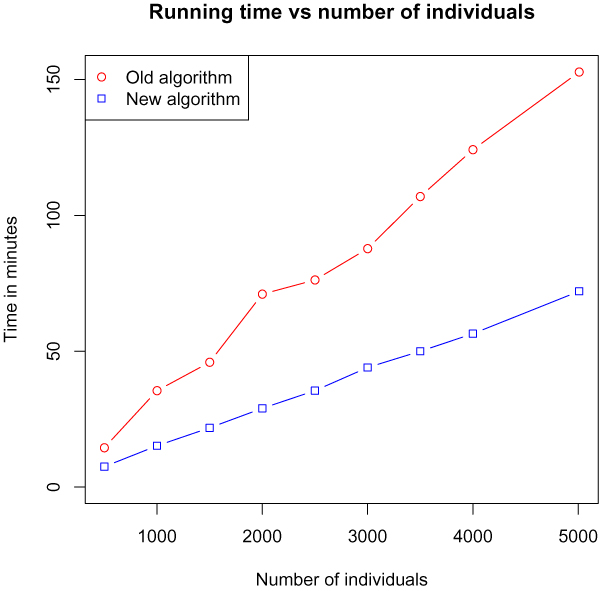
**Time vs. number of individuals**. The time consumption of the two algorithms as a function of the number of individuals in the data sets. The parameters were the default parameters specified in [10] (l = 7, g = 2, s = 2, x = 9).

First we tested the running time as a function of the number of individuals. From the WTCCC data we created test data by picking subsets of individuals, keeping the affected/unaffected ratio constant, and we then ran both algorithms on chromosome 22. Figure 4 shows the "wall time" of both algorithms for varying data sizes. Both algorithms show a linear increase but with the original algorithm having the highest increment.

### Time vs. pattern length

**Figure 5:**
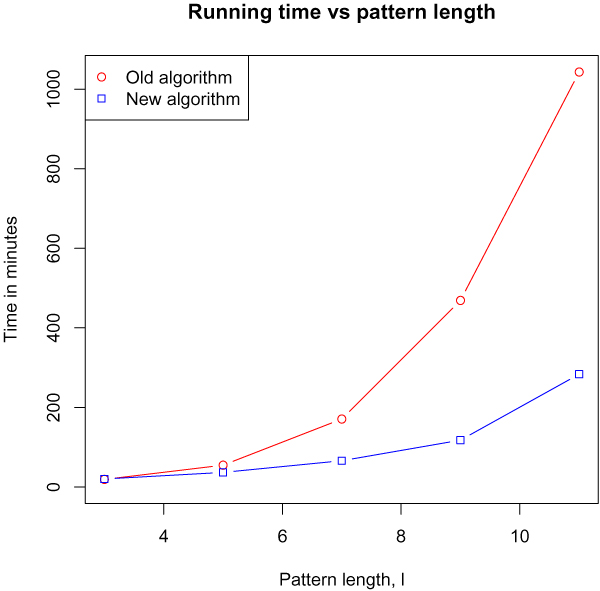
**Time vs. maximal allowed pattern length**. The time consumption of the two algorithms as a function of the maximal allowed pattern length (*l*). The rest of the parameters were fixed at the default settings (g = 2, s = 2, x = 9).

An important parameter for the running time is the maximal allowed pattern length, *l*. Figure 5 show the running time of the two algorithms as a function *l*, when analysing the full chromosome 22 from the WTCCC data set. The running time of both algorithms clearly grows super-linear, but with the time for the new algorithm clearly growing slower.

### Time vs. haplotype diversity

**Figure 6:**
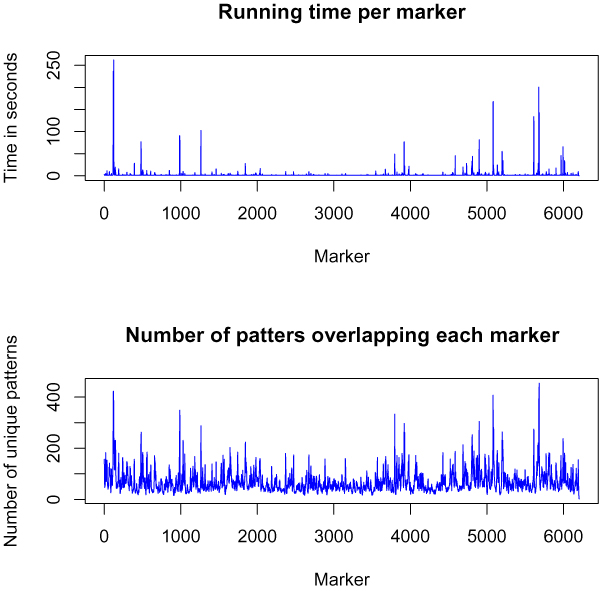
**Running time and number of unique haplotypes per marker**. The time usage per marker on chromosome 22 of the WTCCCC Crohn's disease data (top) and the number of unique haplotypes overlapping each marker (bottom).

**Figure 7:**
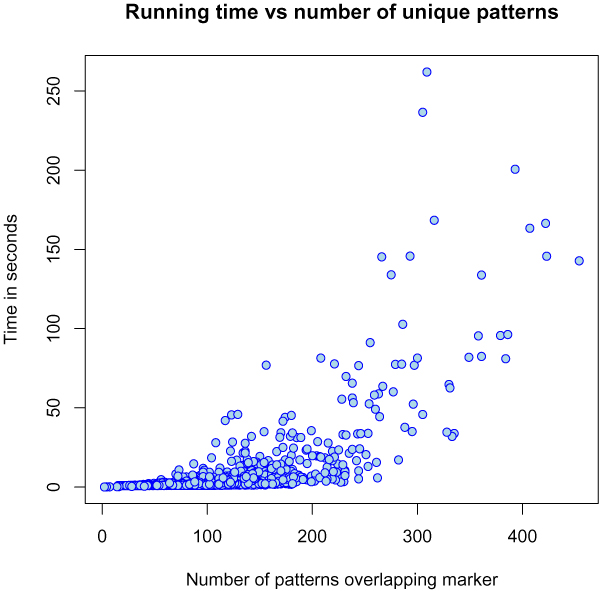
**The relationship between time usage and haplotype diversity**. Time usage per marker vs. number of haplotypes overlapping the marker.

Since the time usage of the new algorithm depends on the number of different haplotypes over a region we expect it to use less time in regions with few distinct haplotypes and more time in regions with many distinct haplotypes. Figure 6 shows the running time (with pattern length *l *= 11) and the number of haplotypes along chromosome 22 of the WTCCC data: The plot on the left shows both the running time per marker (the time to test all patterns beginning in a given marker) together with the number of distinct haplotypes starting in a given marker. Figure 7 shows the running time for scoring a marker as a function of the number of unique haplotypes overlapping the marker.

The same dependency on haplotype diversity is not seen for the old algorithm (results not shown), nor is it expected to be as the old algorithm does not depend on the number of distinct haplotypes seen in the data. Instead, the running time could depend on the maximal score we see when scoring a marker, since this is the threshold used in the branch and bounds heuristic. From the data, however, we do not see a significant effect here.

### Genome-wide analysis

As the final comparison of the old and the new HPM algorithm, we compare the running time on the full Crohn's disease data set. Table 1 shows the time consumption of the two algorithms on each chromosome.

**Table 1:** Time per chromosome. Table showing the time it took to analyze the different chromosomes of the WTCCC Crohn's disease data set. With the following parameters: l = 7, g = 2, s = 2, x = 20.

Crohn's disease running times			
Chromosome	# markers	Original HPM (with optimisation)	New HPM

1	40220	9 hours, 16 minutes	4 hours, 51 minutes
2	41400	9 hours, 37 minutes	4 hours, 54 minutes
3	33799	7 hours, 48 minutes	4 hours, 1 minutes
4	32334	7 hours, 31 minutes	3 hours, 55 minutes
5	32056	7 hours, 20 minutes	3 hours, 59 minutes
6	31470	7 hours, 16 minutes	3 hours, 48 minutes
7	25835	5 hours, 52 minutes	3 hours, 09 minutes
8	27457	6 hours, 11 minutes	3 hours, 21 minutes
9	22864	5 hours, 13 minutes	2 hours, 50 minutes
10	28501	6 hours, 35 minutes	3 hours, 25 minutes
11	26273	6 hours, 8 minutes	3 hours, 11 minutes
12	24954	5 hours, 38 minutes	3 hours, 5 minutes
13	19188	4 hours, 19 minutes	2 hours, 19 minutes
14	15721	3 hours, 37 minutes	1 hour, 54 minutes
15	14355	3 hours, 16 minutes	1 hour, 47 minutes
16	15308	3 hours, 35 minutes	1 hour, 53 minutes
17	11281	2 hours, 37 minutes	1 hour, 23 minutes
18	14881	3 hours, 28 minutes	1 hour, 44 minutes
19	6399	1 hours, 28 minutes	43 minutes
20	12400	2 hours, 53 minutes	1 hour, 23 minutes
21	7125	1 hour, 38 minutes	51 minutes
22	6207	1 hour, 27 minutes	44 minutes

## Conclusion

We have developed a new algorithm for the haplotype pattern mining method and shown that it outperforms the original algorithm on genome wide association data. As a function of the number of individuals or the maximal pattern length, both the new and old algorithm appears to have the same asymptotic running time, with the new algorithm having a significantly smaller time overhead.

The new algorithm is very sensitive to the haplotype diversity. The same is not the case for the old algorithm, but here the mean running time per marker is 8.8 ± 0.57 seconds (with pattern length *l *= 11) where for the new algorithm the mean running time per marker is 2.5 ± 9.6 seconds. It might therefore be worthwhile to use a hybrid algorithm where the new algorithm is used in areas with lower haplotype diversity and the old algorithm is used in areas with high haplotype diversity. If this would reduce the time usage on markers now taking more than 4 seconds to only 3, the hybrid algorithm would spend 1.4 ± 0.63 seconds per marker.

## Competing interests

The authors declare that they have no competing interests.

## Authors' contributions

SB developed the algorithm and implemented the software. All authors designed the experiments. TM and SB draftet the manuscript. All authors read and approved the manuscript.
